# Echocardiography-Guided Percutaneous Patent Ductus Arteriosus Closure: 1-Year Single Center Experience in Indonesia

**DOI:** 10.3389/fcvm.2022.885140

**Published:** 2022-05-23

**Authors:** Sisca Natalia Siagian, Radityo Prakoso, Bayushi Eka Putra, Yovi Kurniawati, Olfi Lelya, Aditya Agita Sembiring, Indriwanto Sakidjan Atmosudigdo, Poppy Surwianti Roebiono, Anna Ulfah Rahajoe, Ganesja Moelia Harimurti, Brian Mendel, Christianto Christianto, Moira Setiawan, Oktavia Lilyasari

**Affiliations:** ^1^Division of Pediatric Cardiology and Congenital Heart Disease, Department of Cardiology and Vascular Medicine, National Cardiovascular Center Harapan Kita, Universitas Indonesia, Jakarta, Indonesia; ^2^Faculty of Medicine, Universitas Indonesia, Jakarta, Indonesia

**Keywords:** congenital heart disease, fluoroscopy, patent ductus arteriosus, percutaneous, echocardiography

## Abstract

**Introduction:**

Since the first successful percutaneous closure under transesophageal echocardiographic (TEE) guidance, many centers explored transcatheter procedures without fluoroscopy. This single-center study is aimed to show the feasibility and safety of percutaneous patent ductus arteriosus (PDA) closure under echocardiography-only guidance during our 1-year experience.

**Methods:**

Patients with PDA were recruited for percutaneous PDA closure guided by either fluoroscopy or echocardiography-only in National Cardiovascular Center Harapan Kita (ClinicalTrials.gov Identifier: NCT05321849, clinicaltrials.gov/ct2/show/NCT05321849). Patients were evaluated clinically and radiologically using transthoracic echocardiography (TTE) at 6, 24, and 48 h after the procedure. The primary endpoint was the procedural success. Secondary endpoints were the procedural time and the rate of adverse events.

**Results:**

A total of 60 patients underwent transcatheter PDA closure, 30 patients with fluoroscopy and 30 patients with echocardiography guidance. All patients had successful PDA closure. There were only residual shunts, which were disappeared after follow-up in both groups, but one patient with a fluoroscopy-guided procedure had moderate tricuspid regurgitation with suspected thrombus in the tricuspid valve. The procedural time was not significantly different between the fluoroscopy and echocardiography groups.

## Introduction

Percutaneous patent ductus arteriosus (PDA) closure has become the preferred therapeutic alternative since its introduction more than three decades ago, where it is also recommended by the American Heart Association/American College of Cardiology (AHA/ACC) and the European Society of Cardiology (ESC). Even though the occluder and device delivery systems have been modified throughout the years, fluoroscopy has been utilized as the gold standard for guidance modality (1, 2). With the advancement of technology, it is now feasible to be performed with limited fluoroscopic exposure. However, the detrimental effects of radiation should not be overlooked, especially for both pediatric patients and interventional cardiologists (3, 4). Pediatric patients are more sensitive toward radiation when compared to adult patients, and they have a longer life expectancy, increasing the chances of lifetime attributable risks of radiation (5). Interventional cardiologists are repeatedly exposed to radiation, thus accumulating the risks of radiation (6). Since Ewert et al. reported the first successful transcatheter closure of atrial septal defect (ASD) under transesophageal echocardiographic (TEE) guidance, many centers explored transcatheter procedures without fluoroscopy, such as ours (7). Our center has been routinely performing transcatheter closure under echocardiographic guidance in 220 patients with ASD or ventricular septal defects (VSDs) with satisfactory outcomes (8). Therefore, this single-center study is aimed to show the feasibility and safety of percutaneous PDA closure under echocardiography-only guidance during our 1-year experience.

## Methods

### Study Design and Setting

From March 2019 to April 2020, a total of 60 patients with PDA were recruited as participants for this prospective, single-center, and non-randomized clinical trial. They were divided equally into two groups: the fluoroscopy group, which underwent percutaneous PDA guided by fluoroscopy and the echocardiography group, which underwent percutaneous PDA closures guided by transthoracic echocardiography (TTE) and TEE at the National Cardiovascular Centre Harapan Kita, Jakarta, Indonesia. Patients with stable hemodynamic and clinically asymptomatic PDA were evaluated and considered for transcatheter PDA closure. Significant chamber enlargement and mild to moderate pulmonary hypertension were also the indications for transcatheter closure. Ductal morphology was evaluated using multiplanar TTE and TEE imaging.

After the procedures, patients were evaluated and followed up after 6, 24, and 48 h to observe any residual shunts and complications. This study protocol conforms to the ethical guidelines of the Declaration of Helsinki and the Nuremberg Code with approval from the Research Ethics Committee of National Cardiovascular Center Harapan Kita No LB.02.01/VII/475/KEP076/2020. Written consent to perform the procedure and to use the patients’ medical records for this study was obtained from the patients or their parents.

### Study Population

The inclusion criteria for this study are children under 18 years old with type-A PDA who were planned for PDA closure. We only conducted this treatment in type A patients with PDA in our 1-year experience since, according to the literature, isolated patients with PDA are mostly type A PDA. The exclusion criteria include the coexistence of other associated congenital heart diseases requiring surgical intervention and PDA patients with irreversible high pulmonary vascular resistance (PVR) unreactive with the oxygen test.

### Pre-procedural Preparation and Device Selection

All recruited patients received pre-procedural examinations consisting of a standard electrocardiogram, a chest X-ray, and blood tests. Device size selection was based on the measurement of the diameter of the PDA isthmus during echocardiography assessment. We prefer to utilize Amplatzer Duct Occluder II (AGA) or Konar Multifunctional VSD Occluder (LifeTech) if the PDA is rather small. We utilized Amplatzer Duct Occluder I (AGA), Konar Multifunctional VSD Occluder (LifeTech), or PDA Occluder Device (MemoPart) if the PDA was rather large. We use a device that can be utilized retrogradely or antegradely in specific circumstances where the PDA size is large but can still be conducted retrogradely. The selection of the occluders from the available types, Amplatzer Duct Occluder I (AGA), Amplatzer Duct Occluder II (AGA), PDA Occluder Device (MemoPart), PDA Occluder Device (LifeTech), Konar Multifunctional VSD Occluder (LifeTech), Multifunctional Occluder (LifeTech), and HeartR PDA Occluder (LifeTech), was determined by the operator according to the size of PDA.

### Instrumentation and Percutaneous Approach

**FIGURE 1:**
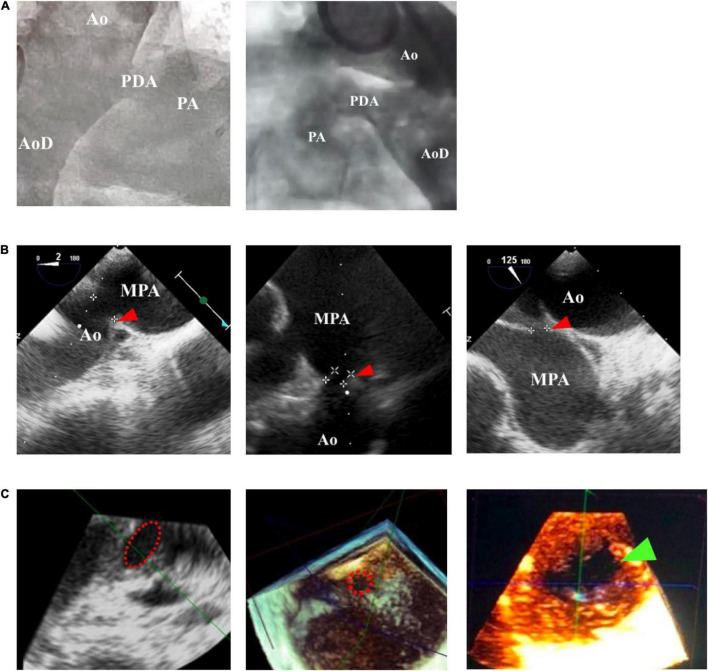
Comparison of patent ductus arteriosus (PDA) size and morphology measurements using fluoroscopy and echocardiography guidance. **(A)** Fluoroscopy can only provide a 2-dimensional image, whereas **(B)** transthoracic echocardiography (TTE) and transesophageal echocardiography (TEE) can provide a 3-dimensional structure as shown by the red arrowhead. **(C)** Cross-sectional PDA morphology measured by using transesophageal echocardiography (TEE), indicated by the dotted red circle. Green arrowhead showed oval-shaped PDA. Ao, aorta; AoD, descending aorta; MPA, main pulmonary artery; PA, pulmonary artery.

**FIGURE 2:**
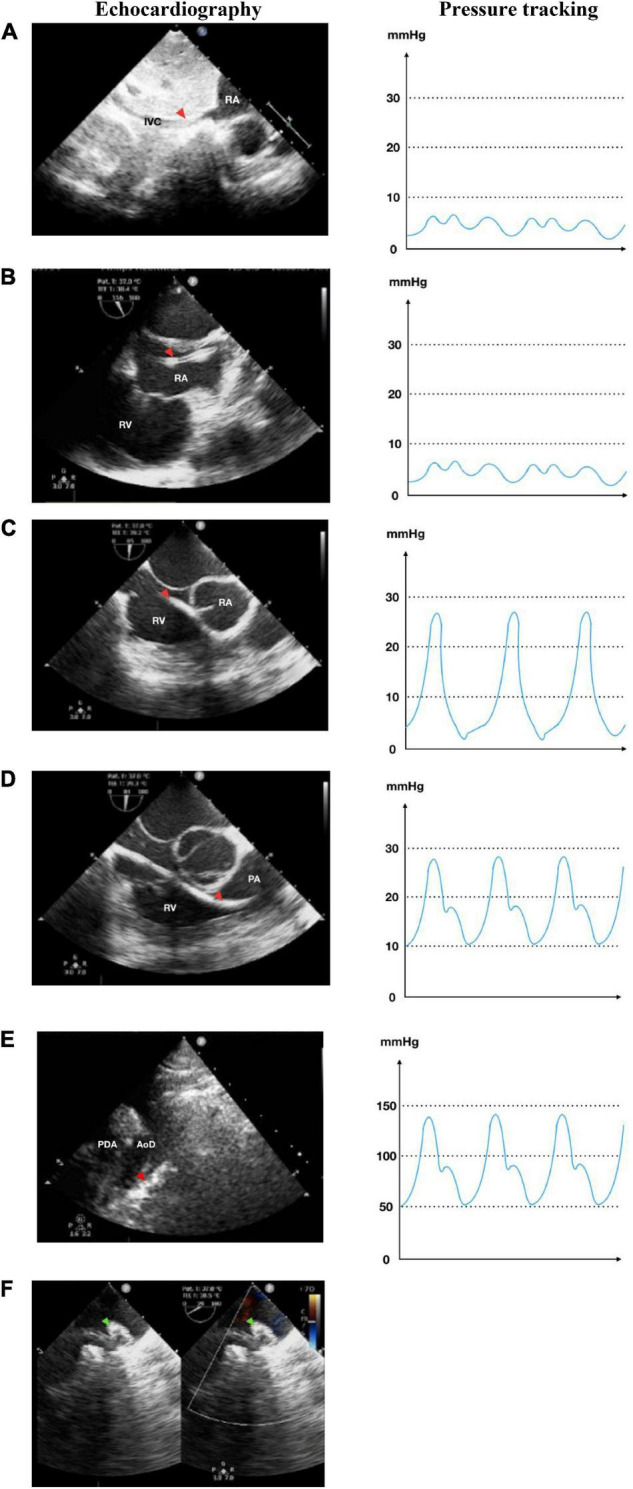
Antegrade transvenous approach of percutaneous patent ductus arteriosus (PDA) closure. **(A)** Catheter is seen in IVC in transthoracic echocardiography (TTE) subxiphoid view. **(B)** Catheter is seen in RA in TEE bicaval view (90–110°). **(C)** Catheter in RV TEE 90° view. **(D)** Catheter enters PA in TEE 90° view. **(E)** Catheter crossed from PA towards AoD through PDA in TTE arch view. **(F)** The device is stowed in place in TEE (40–50°). IVC, inferior vena cava; RA, right atrium; RV, right ventricle; PA, pulmonary artery; Ao, aorta; LA, left atrium; LV, left ventricle; AoD, descending aorta; PDA, patent ductus arteriosus. Red arrowhead shows the catheter head. Green arrowhead indicates the occluder device.

**FIGURE 3:**
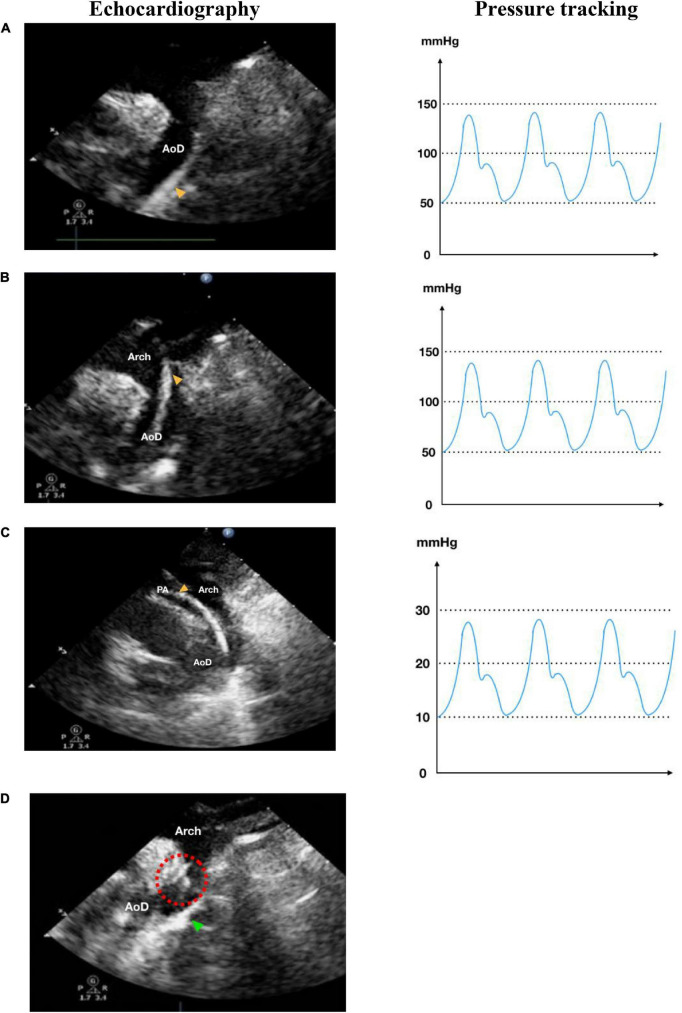
Retrograde transarterial approach of percutaneous patent ductus arteriosus (PDA) closure. **(A)** Catheter is seen in AoD in TTE arch view. **(B)** Catheter is pushed towards the aortic arch in TTE arch view. **(C)** In parasternal short axis TTE view as high as the great arteries, the catheter is seen in PA through PDA from AoD. **(D)** In TTE arch view, the device is stowed in place (shown by red dotted circle) after being delivered from the delivery cable. IVC, inferior vena cava; RA, right atrium; RV, right ventricle; PA, pulmonary artery; Ao, aorta; LA, left atrium; LV, left ventricle; AoD, descending aorta; PDA, patent ductus arteriosus. Orange arrowhead shows the catheter head. Green arrowhead indicates the occluder device.

The patients were sedated under general anesthesia (GA) and intubated with an endotracheal tube. At our institution, all pediatric catheterization procedures are performed under general anesthetic. This is because pediatric patients are sometimes uncooperative and may throw a tantrum during the treatment, complicating the procedure. Evaluation of the morphology and diameter of the PDA isthmus and ampulla were measured with the assistance of TTE high parasternal view and/or TEE high esophageal aortic long-axis view (Figure 1). Asepsis and antisepsis in right and left inguinal regions and local anesthesia with 2% lidocaine injection were performed in the femoral region. Venous or arterial access was made by puncturing the femoral vein or artery. A 4–6 French percutaneous sheath with an introducer set was inserted using the Seldinger technique. For diagnostic and interventional purposes, a 4–6 French Judkins Right (JR) guiding, or multipurpose (MP) catheter was used with a 0.025 or 0.035 standard guidewire. Meanwhile, a 7–12 French JR guiding catheter and delivery sheath were used for device implantation. A stepwise approach to antegrade and retrograde percutaneous PDA closure can be seen in Figures 2, 3. The dose of anticoagulant used in our procedure was heparin 50–100 IU/kgBW. While there is no difference in heparin doses for antegrade and retrograde approaches, we would add additional doses if the duration of the procedure exceeds 1 h. Antibiotic cefazolin was given 50 mg/kgBW IV before the occluder device was implanted. Evaluation of the device position, residual shunt, and turbulence in the aorta and pulmonary artery (PA) was made before and after the device detachment from the delivery cable.

### Follow-Up and Outcome

Patients were evaluated clinically and through imaging by TTE at 6, 24, and 48 h after the procedure. The primary endpoint was the outcome of the procedural success. Successful closure was defined as successful device implantation, without device migration, and no residual shunt or central mild shunt was detected by TTE at the time of post-discharge follow-up.

The secondary endpoints were procedural times and the rate of adverse events. The procedural time was measured from the initiation of GA to the post-procedural evaluation using TTE. Adverse events were defined as any complications, such as occluder migration, hemolysis, peripheral vascular complications, and residual shunt (except central mild shunt), detected during the procedures, immediately after the procedures, or at post-discharge follow-up. Data acquired from the patients’ medical records were patient demographics (age, gender, and weight), the size of the PDA, the type and size of the device, the crossing catheter, and the technical approaches.

### Statistical Analysis

The analysis was performed on an intention to treat basis, with outcomes related to the initial treatment decision. The Shapiro-Wilk test was used as normality test for numerical data where the data were represented as mean (SD) for normally distributed data and median (range) for skewed data. The independent *t*-test was used for comparison of numerical data between the two groups for normally distributed data, while the Mann-Whitney test was used for skewed data. All data analyses were carried out using Statistical Package for Social Science (SPSS) 26.0 software. Values of *p* lower than 0.05 were considered significant.

## Results

This study included 60 patients in total, where 30 patients were allocated to the echocardiography (test) group and 30 patients were allocated to the fluoroscopy (control) group. The summarized baseline characteristics of subjects and the approaches used, time taken for the procedures, crossing catheter, and devices used can be seen in Table 1.

**TABLE 1:** Summary of baseline characteristics of patients and procedures.

Variables	Echocardiography group (*n* = 30)	Fluoroscopy group (*n* = 30)	*P*-value
Age, years	6.0 (2.4–9.0)	2.8 (1.8–20.3)	0.942
Sex (male/female)	14/16	6/24	
Weight, kg	17.0 (12.2–23.0)	12.9 (8.0–40.3)	0.554
PDA size, mm	5.07 ± 2.25	5.58 ± 1.97	0.465
**Approach**			
Retrograde	23	8	
Antegrade	7	22	
Procedural time	50 (25–58)	53 (36–77)	0.090
Retrograde	28.5 (20.3–48.3)	36 (34–59.5)	0.113
Antegrade	61 (53–80)	61.5 (36.5–79.8)	0.575
**Crossing catheter**			
JR guiding catheter	30	6	
Multipurpose guiding catheter	0	24	
**Device type**			
PDA Occluder Device (Memopart)	1	16	
PDA Occluder Device (LifeTech)	4	0	
Konar MF VSD Occluder (Lifetech)	8	8	
HeartR PDA Occluder (Lifetech)	0	3	
ADO I (AGA)	0	2	
ADO II (AGA)	10	1	
MF Occluder (LifeTech)	7	0	
Device size, mm	13.4 ± 3.5	16.5 ± 3.7	0.920

*PDA, patent ductus arteriosus; JR, Judkins Right; MF, Multifunctional; VSD, ventricular septal defect; ADO, Amplatzer Duct Occluder.*

### Study Primary Endpoint

All the procedures were successful with no device dislodgement in both groups. From the echocardiography group, 13 out of 30 patients had mild residual central shunt, which was eventually resolved during follow-up. On the other hand, there were 17 out of 30 patients with mild residual central shunt after the procedures in which fluoroscopy was used, which was also resolved during follow-up. From these results, we can conclude that the echocardiography group is non-inferior to the fluoroscopy group.

### Study Secondary Endpoint

The procedures were conducted using either antegrade approach or retrograde approach. The median procedural time of using an antegrade approach for the test and control group was 28.5 (20.3–48.3) and 36 (34–59.5) min, respectively (*p* = 0.113), while the median procedural time of using a retrograde approach for the test and control group was 61 (53–80) and 61.5 (36.5–79.8) min, respectively (*p* = 0.575). Without taking into account the approach, the procedural time was not significantly different in the echocardiography group and the fluoroscopy group [50 (25–58) min vs. 53 (36–77), *p* = 0.09].

During the procedure, after the procedure, and after discharge of the patients, there were no adverse events, such as major procedural complications (death, peripheral vascular injury, pericardial effusion, and pericardial tamponade). All patients neither required blood transfusion nor any inotropic support. An echocardiographic assessment conducted during follow-up showed complete closure in all patients with no left PA stenosis and aortic arch obstruction identified. All patients had successful closures with no residual shunts and recovered well. There were no post-procedural unresolved residual shunts, thrombosis, or any valvular regurgitations observed during follow-up, except in one patient from the fluoroscopy group who had moderate tricuspid regurgitation with suspected thrombus in the tricuspid valve, which was detected during follow-up.

## Discussion

Transcatheter closure has become the main approach for most PDA closures as it has lower complications and shorter hospital stay when compared to the surgical approach (1, 9). The 2018 AHA/ACC and the 2020 ESC guidelines for the management of congenital heart disease recommend transcatheter closure for all PDAs with evidence of LV volume overload regardless of symptoms, except in patients with Eisenmenger physiology, lower limb desaturation on exercise, PA systolic pressure greater than two-thirds of systemic systolic pressure or PVR greater than two-thirds of systemic vascular resistance adults (10, 11). For the last four decades, transcatheter PDA closure under fluoroscopy guidance has been the most popular choice due to its convenience as it can localize the wire and device accurately (12). Since the success of the first echocardiography-guided balloon atrial septostomy, which was performed by Rashkind in 1966 (13), many cardiologists have been experimenting with using echocardiography as guidance for many procedures, such as PDA closure (8, 14).

The frequent use of fluoroscopy may cause long-term side effects, depending on the dosage and duration of radiation exposure. In addition, the complexity of a procedure also adds to the duration of exposure, as in transcatheter PDA closure (15). Prolonged radiation not only harms pediatric patients who are more sensitive to radiation than adults (5), but it is also a risk to interventionists, especially those who routinely perform these procedures. While the advancement of technology allows for minimal fluoroscopic exposure through non-ionic contrast, the accumulated exposure on interventionists should not be overlooked (6), where side effects may show as late as the 70–90 s (16). Studies have shown that radiation increases the risks of skin injuries, cataracts, hair loss, and neoplasms (17). A study by Roguin et al. reported 44 interventional cardiologists with brain and neck cancer on their left side as it is exposed to more radiation than the right (18, 19).

In this study, we compared the time taken for PDA closure procedures using fluoroscopy and echocardiography. The median procedural time of using both antegrade and retrograde approaches for the test and control groups was insignificant (*p* = 0.575). The retrograde approach had a shorter procedural time in comparison to the antegrade approach where the catheter needs more manipulation to be directed into the right ventricle and PA to reach the PDA (see Figures 2C,D). However, the retrograde approach is limited to patients with low body weight or large PDA since complications might occur through arterial access, such as thrombosis at the access site. Regardless of the approach, the difference in procedural time between the echocardiography group and the fluoroscopy group was insignificant (*p* = 0.09). These results mean that using only echocardiography did not compromise procedural time.

Binobaidan et al. (12), Cao et al. (20), and Ye et al. (21) showed success rates of 100% in PDA closure, while only Zhang et al. (22) showed a success rate of 97.1%. These results were similar to our study in which all the procedures were successful in both echocardiography and fluoroscopy groups. Most studies showed no residual shunt and severe complications after 1 month to 2 years of follow-up (12, 20–22). Pan et al. had 8 cases of small residual shunts in 24 h but resolved afterward (6). The minor complications in these studies include device embolization, acute occluder dislodgement, and small residual shunts (6, 12, 20–22). In our study, there were no complications following echocardiography-guided PDA closures with most of the patients only experiencing residual shunts, which disappeared during follow-up. However, one fluoroscopy-guided procedure resulted in moderate tricuspid regurgitation with a thrombus in the tricuspid valve. This may be due to variable confounding factors, such as the complexity of the PDA, operator experience, or the length of the procedure, which may contribute to the complications despite better visualization in the fluoroscopy group. These results showed that the echocardiography-guided procedure is not inferior to the fluoroscopy-guided procedure.

Based on pre-catheterization data, TTE or TEE showed similarities in assessing the defect size. Shyu et al. showed that TEE provided greater sensitivity to TTE in confirming PDA size (97 vs. 42%) (23). The selection of devices in 38 subjects is based on the size of the PDA. The addition of 2–4 mm from the diameter of the smallest PDA was done in device selection, but the device size would be twice the size of the PDA if it presents with pulmonary hypertension (24). Devices, such as Amplatzer Duct Occluder (ADO II) and Multifunctional (MF) Occluder, could be performed both antegradely and retrogradely. However, ADO II could only be used in patients with small PDAs. MF Occluder is now a better choice since it could be used on a large size PDA with a smaller delivery sheath. In our center, the post-procedural residual shunt is commonly found when using MF Occluder, which disappeared within 24–48 h, possibly due to the soft nature of the device. We recommend choosing devices according to the type and size of the PDA, availability, and convenience of the operators (1).

In comparison to fluoroscopic guidance, the largest hurdle of echocardiography-guided closure is to track the guidewire, catheter, and delivery sheath in the two-dimensional echocardiographic view plane, which is related to safety and procedural time. Conventional intervention techniques emphasize simplicity to determine the catheter location as fluoroscopy detects it through projection. A vast understanding of cardiac anatomy and physiology, along with the utilization of the appropriate guidewire, catheter, delivery sheath, and the positioning of the ultrasound probe, would exceedingly increase accuracy. Interventionists with insufficient experience should go for the standard procedure. Future efforts should be directed toward pre-procedural selection criteria for echocardiography-guided transcatheter PDA closure with devices based on the PDA size and surrounding structures.

## Limitation

The small number of subjects is an unavoidable limitation as this is the first pilot study of echocardiography-guided PDA closure to be conducted in Indonesia. The procedures were conducted by different operators, which may affect results concerning procedural times and complications as the procedures were highly dependent on the operators. Therefore, we recommend further studies with more subjects and skilled operators to validate the feasibility and safety of echocardiography-guided PDA closure.

## Conclusion

Echocardiography-guided PDA closure is non-inferior, even with a limited window view when compared to using fluoroscopy. The procedural time for echocardiography-guided PDA closure was also not significantly different from fluoroscopy-guided PDA closure, which means that using only echocardiography did not compromise procedural time. However, as this is a new technique, this method is especially operator dependent and requires an experienced team for it to be performed successfully. Unfortunately, it would still require years for this method to be widely used.

## Data Availability Statement

The original contributions presented in the study are included in the article/supplementary material, further inquiries can be directed to the corresponding author.

## Ethics Statement

The studies involving human participants were reviewed and approved by the Research Ethics Committee of National Cardiovascular Center Harapan Kita. Written informed consent to participate in this study was provided by the participants’ legal guardian/next of kin.

## Author Contributions

SS conceived the original idea of the manuscript. SS, RP, BP, YK, OLe, AS, IA, PR, AR, GH, and OLi contributed in collecting data and writing the main text of the manuscript. BM, CC, and MS performed statistical analyses and also helped in writing the manuscript. All authors discussed and agreed with the idea of the manuscript and accepted the proofread manuscript.

## Conflict of Interest

The authors declare that the research was conducted in the absence of any commercial or financial relationships that could be construed as a potential conflict of interest.

## Publisher’s Note

All claims expressed in this article are solely those of the authors and do not necessarily represent those of their affiliated organizations, or those of the publisher, the editors and the reviewers. Any product that may be evaluated in this article, or claim that may be made by its manufacturer, is not guaranteed or endorsed by the publisher.
